# Extra Uterine Foetation

**Published:** 1881-08

**Authors:** H. Rozencrans

**Affiliations:** Elgin, Ill.


### ARTICLE V.

**EXTRA UTERINE FŒTATION.** By H. ROZENCRANS, M.D., Elgin, Ill.

On the 28th day of July, 1880, I was called twenty miles into the country to see a sick woman, a Mrs. J., aged 28 years, the mother of two living children. I found this woman suffering severely, and was informed that for three nights she had not slept. Her sufferings were referred mostly to the lower part of her back. I administered to her tincture of opium, and bromide of potassium, a liberal dose; this caused her to sleep most of the night. Examination of this woman satisfied me that she was a subject for an operation; that it would not admit of delay, as she was then very much emaciated; could not retain food, could not rest night nor day, because of great suffering. I advised her to be put on an easy spring wagon, on a mattress, and carefully brought to town for treatment. This was done. I then made a thorough examination of the case, and diagnosticated it a case of extra-uterine fœtation, and from the history of the case to be of about one year duration.

As there was evidently a considerable quantity of fluid in the abdominal cavity, and to satisfy myself more fully of the correctness of my diagnosis, I resolved to introduce the trochar, and did so, about one inch below the navel, on the median line, and drew off at least two gallons of a dark heavy fluid. I then made further examination and could distinctly feel a solid substance in the abdominal cavity and pronounced it a fœtus. I wrote down my diagnosis, and sent it to a physician living in a neighboring town (Victoria), fifty miles away, inviting him and two others to come down and assist me in a operation for the removal of an extra uterine fœtus by abdominal section. Dr. Sutherland and Dr. Blake, the former of Victoria and the latter of Cueso, finally came down and assisted me in the operation, on the 20th day of August, 1880, in Indianola. I, after getting this patient in readiness, administered to her chloroform, and immediately cut down through the abdominal walls on the median line extending from one inch below the umbilicus to near the pubic arch. After cutting through the walls of the abdomen, bringing to view the cyst, I had the patient turned over on her side and at once cut open the cyst, from which flowed freely a considerable quantity of the same character of fluid that I had removed a few days previously with the use of the trochar. I then at once thrust my hand into the sac and grappled the fœtus and at once removed it, feet foremost, dead, and weighing eight pounds. I then tore away the placental mass, and proceeded to the removal of the sac. This I found a difficult thing to do because of its numerous attachments ; portions of it I cut away, portions of it I tore away; a portion of the omentum was removed with it. The right ovary was removed, after ligaturing the pedicle with a cat-gut ligature. The wound was then closed, leaving an opening at its most dependent part, and a tube introdubed for drainage, from whence for many days a copious discharge of putrid matter flowed. To obviate internal bleeding I, immediately after closing the wound, placed two bricks on the abdomen for pressure, leaving them there for half an hour and then removing them. During the operation I used the carbolic spray and diligently, during the after treatment, kept injecting daily into the wound, through the tube, a solution of five per cent. of carbolic acid. The weather was very warm, averaging 95°. I applied pounded ice constantly for the space of three weeks to the abdomen, and for that length of time gave a quinine pill three times a day, of two grs. each. Ordered a milk diet. I drew off her urine twice a day for a week, after that she voided it herself. Her first evacuation from the bowels was on the fifth day. After the operation I never gave her any cathartic medicine. She never suffered any pain after the operation ; the temperature of her body remained quite normal, her pulse was usually one hundred in the morning and would rise to one hundred and twenty in the after part of the day, for about three weeks. I consider that the constant application of ice prevented peritonitis, and with the diligent use of carbolic acid prevented pyæmia or blood poisoning. I discharged my patient cured, on the 6th day of November, seventy-eight days after the operation. This operation was performed on the 20th day of August, 1880, in the Town of Indianola, State of Texas. I was one hour and a half in performing it. I am not aware that a similar operation was ever before performed in that State

June, 1881.

